# Navigating the Diagnosis and Management of Papillary Muscle Rupture Following Inferior Myocardial Infarction

**DOI:** 10.7759/cureus.103928

**Published:** 2026-02-19

**Authors:** Aishwarya Sharma, Haroon Mujahid, Amir Darki

**Affiliations:** 1 Internal Medicine, Loyola University Medical Center, Maywood, USA; 2 Cardiology, Loyola University Medical Center, Maywood, USA

**Keywords:** acute mitral regurgitation, cardiogenic shock, echocardiography, inferior myocardial infarction, papillary muscle rupture

## Abstract

Papillary muscle rupture (PMR) is often underdiagnosed or diagnosed late due to its low incidence and nonspecific symptoms in acutely presenting, critically ill patients. This report presents a case of a 74-year-old male who developed acute mitral regurgitation secondary to PMR in the context of an initially conservatively managed inferior wall infarction following recent spine surgery. The discussion highlights the most appropriate diagnostic approach for critically ill patients presenting with acute hypoxic respiratory failure in the cardiac ICU. It also examines optimal management strategies for PMR in the setting of cardiogenic shock and pulmonary edema, emphasizing the importance of timely recognition and targeted intervention.

## Introduction

Papillary muscle rupture (PMR) is a rare but catastrophic mechanical complication of acute myocardial infarction (MI), occurring in less than 1% of cases and associated with exceedingly high mortality if not promptly recognized and treated [1]. It most commonly follows inferior MI due to the often solitary blood supply of the posteromedial papillary muscle, rendering it particularly vulnerable to ischemic necrosis [2].

PMR leads to acute, severe mitral regurgitation (MR), causing an abrupt rise in left atrial and pulmonary venous pressures. Unlike chronic MR, where the heart has time to undergo compensatory remodeling, acute MR from PMR overwhelms the left atrium and ventricle with sudden volume overload, leaving no time for compensatory dilatation and resulting in reduced stroke volume. This can trigger flash pulmonary edema and subsequent cardiogenic shock. The hemodynamic collapse that follows is often dramatic and requires immediate intervention.

Prompt diagnosis relies on echocardiography, with transesophageal echocardiography (TEE) offering superior sensitivity for identifying partial or complete rupture [3]. Definitive management has traditionally required urgent surgical mitral valve repair or replacement; however, perioperative mortality remains high, particularly in patients presenting with hemodynamic instability or significant comorbidities [3].

In recent years, advances in mechanical circulatory support (MCS) and transcatheter mitral interventions have expanded potential stabilization and treatment strategies for select high-risk patients [4]. These data suggest that early left ventricular (LV) unloading should be considered when feasible in PMR-associated shock, particularly as a bridge to definitive surgery. Furthermore, rupture morphology is clinically relevant: partial PMR may permit limited leaflet support and temporizing interventions, whereas complete rupture causes sudden loss of valve competence and requires urgent surgical replacement.

We report a case of delayed inferior ST-segment elevation MI (STEMI) complicated by acute PMR and cardiogenic shock. This case highlights the diagnostic and therapeutic complexities of late-presenting STEMI, particularly when recent spinal surgery precludes the use of systemic anticoagulation and urgent revascularization. It also illustrates how patients who initially appear clinically stable may experience rapid and progressive hemodynamic collapse. Delayed MI presentations require heightened vigilance, as competing procedural risks can significantly influence the timing of intervention, choice of MCS, and overall outcomes in the setting of acute mechanical complications of MI.

## Case presentation

**Figure 1:**
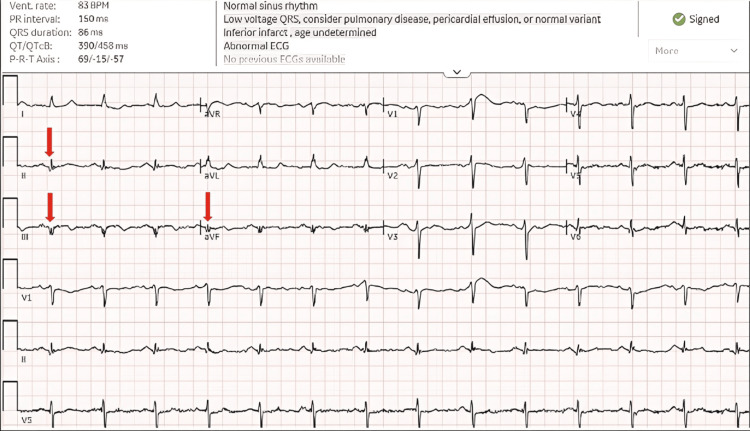
Normal sinus rhythm with low-voltage QRS and Q waves in leads II, III, and aVF (red arrows)

A 74-year-old male with a past medical history of coronary artery disease status post percutaneous coronary intervention to the right coronary artery (RCA) in 2006, hypertension, deep vein thrombosis (on apixaban), chronic obstructive pulmonary disease, obstructive sleep apnea, and a recent L2-L4 laminectomy presented 10 days after surgery with chest pain. Laboratory evaluation revealed markedly elevated high-sensitivity troponin (HST) at 7,927 ng/L (normal range 0-20 ng/L). ECG demonstrated new Q waves in leads II, III, and aVF, consistent with acute inferior infarction, which were absent on the preoperative ECG (Figure 1).

**Figure 2:**
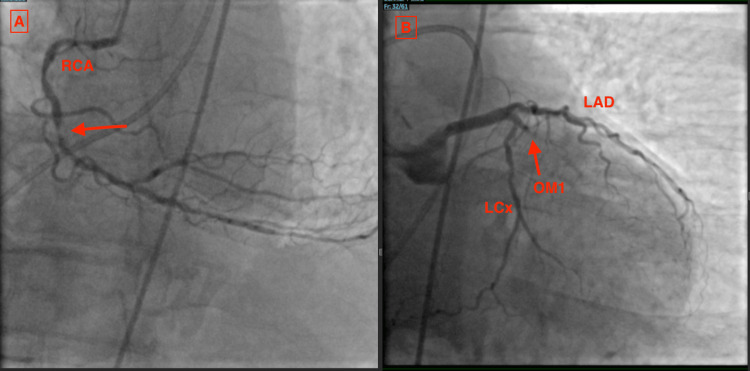
Right (A) and left (B) coronary arteries in RAO caudal view Red arrows indicate in-stent restenosis of the RCA (A) and 100% occlusion of OM1 (B). Moderate disease is noted in the LAD and LCx arteries (B). LAD, left anterior descending; LCx, left circumflex; OM1, obtuse marginal 1; RAO, right anterior oblique; RCA, right coronary artery

Left heart catheterization revealed 90% in-stent restenosis of the RCA and 100% occlusion of the obtuse marginal artery (Figure 2).

Right heart catheterization (RHC) was performed to further evaluate hemodynamic filling pressures and the potential need for MCS. RHC demonstrated elevated pre- and postcapillary filling pressures (mmHg) (RA 11, RV 55/12, PA 55/22/31, and PCWP 22) with preserved cardiac output and index (6.7 L/min/3.1 L/min/m²), so MCS was initially deferred. Transthoracic echocardiography revealed a new reduction in LV ejection fraction to 35%, a dilated left ventricle, and inferolateral wall hypokinesis. Right ventricular systolic function appeared preserved by echocardiogram (TAPSE 2.7 cm, tissue Doppler imaging 30 cm/s) and catheterization-based hemodynamic parameters (pulmonary artery pulsatility index ~3). No significant valvular abnormalities were noted at that time.

Given the patient’s overall hemodynamic stability in the setting of a late-presenting STEMI, coronary intervention was deferred, and a multidisciplinary discussion was undertaken regarding revascularization. The neurosurgery team expressed substantial concern regarding postoperative spinal epidural hemorrhage and potential neurologic compromise associated with systemic anticoagulation and dual antiplatelet therapy (DAPT) after a recent laminectomy performed 10 days earlier. Accordingly, following shared decision-making with the patient, coronary revascularization was deferred for seven days.

On hospital day 3, the patient developed acute hypoxic respiratory failure and hypotension, requiring emergent intubation and vasopressor support. Laboratory findings demonstrated elevated lactate (5.5 mmol/L), HST 2,600 ng/L, and creatinine 1.2 mg/dL, reflecting progression to cardiogenic shock. A bedside point-of-care ultrasound performed by a fellow revealed new, acute severe MR with visualization of a ruptured papillary muscle, a large flail gap, and vena contracta >1 cm, suggestive of acute PMR, as well as reduced right ventricular systolic function (TAPSE 1 cm). A stat chest X-ray showed increased central pulmonary vascularity with new, diffuse bilateral pulmonary edema.

**Figure 3:**
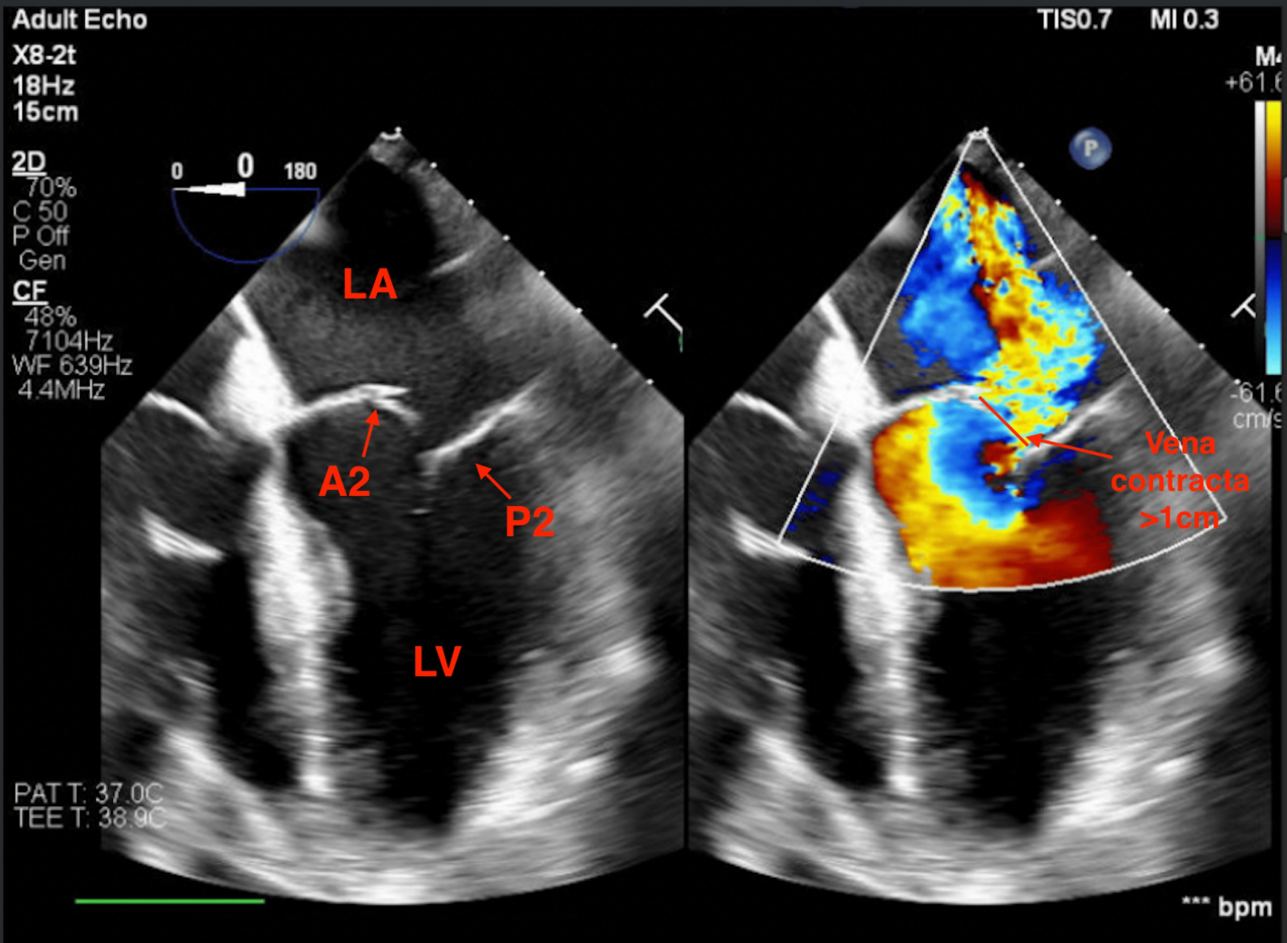
TEE, four-chamber view, showing severe MR A2, anterior mitral leaflet 2 segment; LA, left atrium; LV, left ventricle; MR, mitral regurgitation; P2, posterior mitral leaflet 2 segment; TEE, transesophageal echocardiogram

The patient was urgently taken to the cardiac catheterization laboratory and underwent TEE, which confirmed severe MR due to PMR (Figure 3). Notably, there was a flail of the A1/A2 segments with a posteriorly directed MR jet, consistent with acute severe MR physiology.

The patient was started on IV nitroprusside and IV furosemide for acute afterload and preload reduction. Repeat RHC revealed severely elevated pre- and postcapillary filling pressures (mmHg) (RA 22, RV 66/24, PA 73/38/53, and PCWP 41) and severely reduced cardiac output/cardiac index (CO/CI 3.3 L/min/1.5 L/min/m²), consistent with severe cardiogenic shock (Table 1).

**Table 1:** Laboratory and hemodynamic parameters across clinical course HD, hospital day; HST, high-sensitivity troponin; PA, pulmonary artery; PCWP, pulmonary capillary wedge pressure; RA, right atrial; RV, right ventricular

Parameter	Normal range	Initial presentation	HD 3 (acute decompensation)
Lactate (mmol/L)	0.5-2.0	1.1	5.5
HST (ng/L)	<20	7,927	2,600
Serum creatinine (mg/dL)	0.6-1.3	0.69	1.2
RA pressure (mmHg)	2-6	11	22
RV pressure (mmHg)	15-30/2-8	55/12	66/24
PA pressure (mmHg)	15-30/8-15 (mean 10-20)	55/22 (mean 31)	73/38 (mean 53)
PCWP (mmHg)	6-12	22	41
Fick cardiac output (L/min)	4.0-8.0	6.7	3.3
Cardiac index (L/min/m²)	2.5-4.0	3.1	1.5

An intra-aortic balloon pump (IABP) was placed for MCS due to its rapid deployment and ease of insertion. Cardiothoracic surgery was emergently consulted; the in-house team evaluated the patient in the catheterization laboratory, and the operating room was mobilized for immediate surgical intervention.

**Figure 4:**
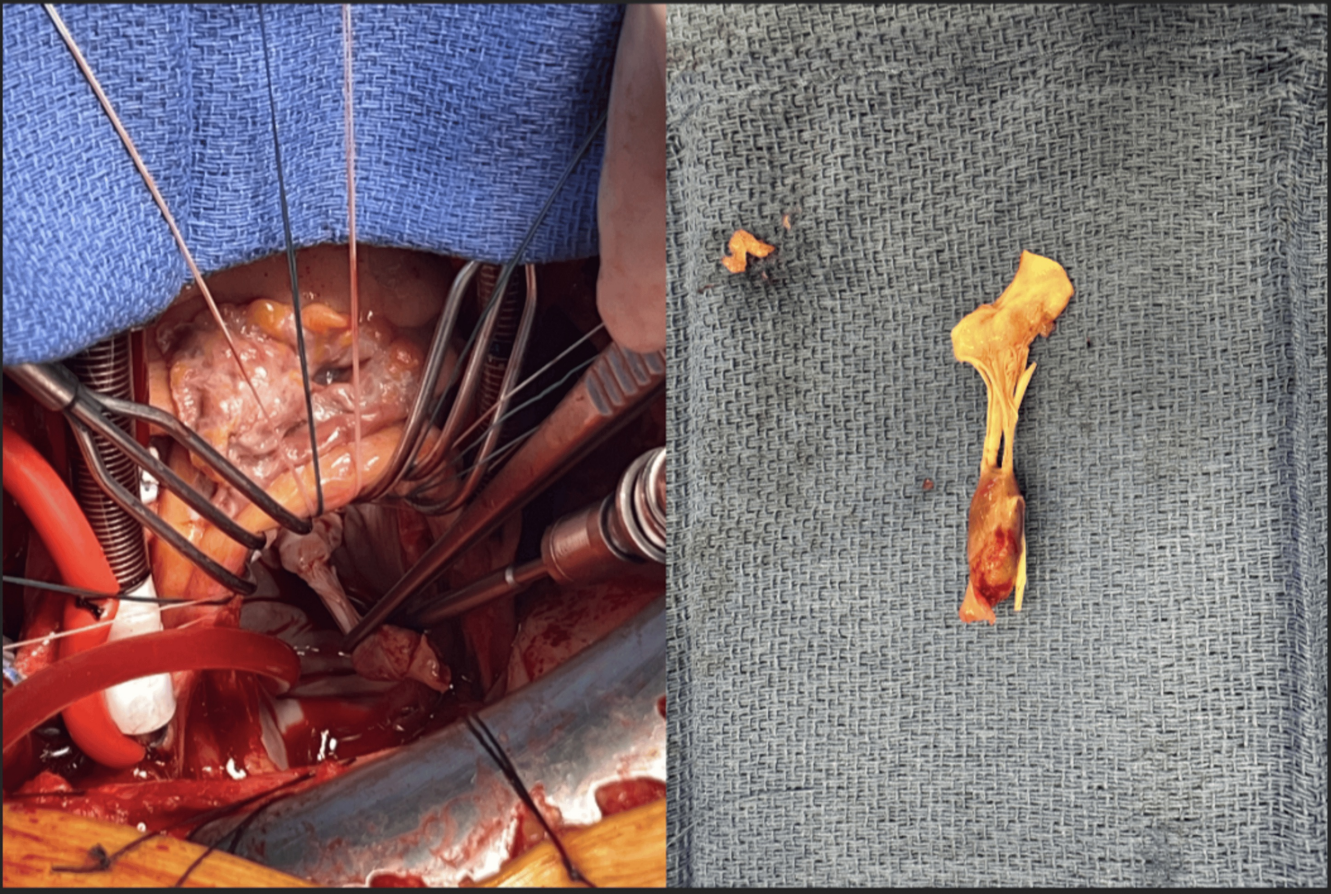
Intraoperative (left) and gross specimen (right) images of the ruptured papillary muscle segment of the anterior leaflet

In the operating room, the patient underwent excision of the ruptured papillary muscle (Figure 4) with bioprosthetic mitral valve replacement (33 mm St. Jude) using chordal preservation via the posterior mitral leaflet preservation technique. He also underwent single-vessel coronary artery bypass grafting (saphenous vein graft to the posterior descending artery) and left atrial appendage occlusion. The patient tolerated 133 minutes of extracorporeal circulation and remained hemodynamically stable in the immediate perioperative period.

However, his subsequent hospital course was complicated by septic shock secondary to hospital-acquired pneumonia, requiring triple vasopressor support; ventilator-dependent acute-on-chronic respiratory failure ultimately necessitating tracheostomy; sternal wound dehiscence; and intermittent episodes of atrial fibrillation and flutter with rapid ventricular response. Therapeutic heparin was initiated due to early findings of diminished Doppler signals concerning impaired arterial perfusion. The patient subsequently developed heparin-induced thrombocytopenia and thrombosis, complicated by bilateral limb ischemia and forearm compartment syndrome, necessitating bilateral decompressive fasciotomies and open thrombectomy for management of his hypercoagulable state and associated thrombosis.

Given persistent dependence on vasoactive support, inability to wean ventilatory support, and the anticipated need for bilateral hand amputations due to critical limb ischemia, the patient’s prognosis was deemed poor. After discussions with the multidisciplinary care team and the patient’s family, a decision was made to transition to comfort-focused care, and the patient subsequently died.

## Discussion

This case highlights the potential for rapid hemodynamic collapse following acute PMR after inferior MI and emphasizes the importance of early diagnosis, timely hemodynamic stabilization, and coordinated management strategies. Early coronary revascularization following MI is associated with lower rates of subsequent MR, whereas delayed revascularization increases the risk of mechanical complications. Severe MR occurs in approximately 0.7-2.8% of patients undergoing primary percutaneous coronary intervention, with a higher incidence reported when revascularization is delayed beyond 72 hours, particularly in non-STEMI [5]. In the present case, while these risks were recognized, definitive revascularization was deferred due to the prohibitive risk of catastrophic spinal hemorrhage associated with systemic anticoagulation and DAPT shortly after laminectomy. Temporizing strategies, such as limited or balloon-only angioplasty, may be considered in select scenarios but must be balanced against bleeding risk and overall clinical stability.

The risk of PMR is especially pronounced in inferior MI, which disproportionately affects the posteromedial papillary muscle owing to its single coronary blood supply, rendering it particularly vulnerable to ischemic necrosis and rupture. Prompt echocardiographic diagnosis is critical, as mortality approaches 80-90% without intervention [3]. While transthoracic echocardiography may suggest acute severe MR, TEE offers superior sensitivity and specificity for identifying papillary muscle disruption. Pino et al. emphasized that direct visualization of a partially or completely detached papillary muscle head appearing as a hypermobile echodensity within the left ventricle is the most definitive echocardiographic finding of PMR, often accompanied by leaflet flail and severe eccentric MR [3].

Echocardiographic evidence of leaflet flail and papillary muscle disruption distinguishes PMR from ischemic functional MR (iMR). While the management of chronic or gradually progressive iMR associated with annular dilation remains an area of clinical uncertainty, acute PMR is widely regarded as a surgical emergency because it produces sudden severe MR, leading to pulmonary edema and cardiogenic shock in the absence of adaptive remodeling [6].

An important determinant of therapeutic approach is whether PMR is partial or complete. Partial rupture preserves some degree of papillary muscle continuity, allowing residual leaflet support and limited coaptation. This anatomical substrate has enabled the use of transcatheter edge-to-edge repair (TEER) in carefully selected high-risk patients [7]. Haberman et al. reported a multicenter experience demonstrating that TEER can effectively reduce MR and stabilize hemodynamics in patients with papillary muscle injury complicating acute MI, particularly when surgical risk is prohibitive [8]. Similarly, Yamamoto et al. described successful transcatheter stabilization in a frail elderly patient with partial PMR, supporting the feasibility of TEER as salvage or bridge therapy in select cases [9]. In contrast, complete PMR results in total detachment of the papillary muscle head, producing a freely mobile leaflet without tethering. In these cases, TEER is generally not feasible due to the inability to adequately grasp and approximate the leaflets, and urgent surgical mitral valve replacement remains the standard of care.

Patients with acute PMR frequently present in cardiogenic shock, necessitating rapid preoperative stabilization to optimize end-organ perfusion [10]. While vasopressors and afterload reduction may provide transient support, they do not address the profound volume overload and regurgitant physiology of acute MR. Mechanical LV unloading has emerged as a critical adjunct in this setting. Although the DanGer Shock trial demonstrated a mortality benefit with early Impella CP use in infarct-related cardiogenic shock, patients with mechanical complications such as PMR were excluded, limiting direct applicability [11].

Nonetheless, growing evidence supports LV unloading in PMR. Iino et al. reported successful hemodynamic stabilization using Impella 5.5 following emergency surgery for mechanical complications of MI, highlighting its role in reducing LV filling pressures and improving forward flow [12]. Rekhtman et al. further demonstrated that preoperative optimization with Impella 5.5 can improve surgical candidacy and hemodynamic stability in patients with ruptured papillary muscle [13].

In contrast, although IABP support alone has the advantage of rapid placement, it provides limited unloading in the setting of torrential MR, and venoarterial extracorporeal membrane oxygenation without LV unloading may worsen LV distension and pulmonary congestion [14]. Proceeding directly to surgery without stabilization carries a high operative risk, particularly in patients presenting with shock.

Despite advances in surgical techniques, mechanical support, and transcatheter therapies, PMR remains associated with high morbidity and mortality. A systematic review and meta-analysis by Massimi et al. demonstrated persistently high operative mortality rates, particularly among patients presenting with cardiogenic shock or multiorgan dysfunction [15]. Our patient’s postoperative course was complicated by recurrent shock, respiratory failure, arrhythmias, and limb ischemia, ultimately leading to a decision for comfort-focused care. This underscores the sobering reality that even with contemporary management, outcomes remain poor in elderly patients with delayed MI presentation and multiple comorbidities.

## Conclusions

Acute PMR is a devastating mechanical complication of MI that demands immediate recognition and intervention. TEE plays a pivotal role in diagnosis and in distinguishing partial from complete rupture, as rupture morphology directly informs therapeutic options. While urgent surgical mitral valve replacement remains the definitive therapy, contemporary strategies incorporating MCS and, in select cases, TEER can provide critical hemodynamic stabilization in high-risk patients. This case underscores the importance of maintaining early suspicion in patients with sudden pulmonary edema or shock following inferior MI, timely echocardiographic evaluation, and multidisciplinary, patient-centered decision-making. Despite advances in care, outcomes remain poor, highlighting the need for rapid diagnosis, individualized management, and early goals-of-care discussions in this critically ill population.
